# Discovery of the first hydrothermal field along the 500-km-long Knipovich Ridge offshore Svalbard (the Jøtul field)

**DOI:** 10.1038/s41598-024-60802-3

**Published:** 2024-05-03

**Authors:** Gerhard Bohrmann, Katharina Streuff, Miriam Römer, Stig-Morten Knutsen, Daniel Smrzka, Jan Kleint, Aaron Röhler, Thomas Pape, Nils Rune Sandstå, Charlotte Kleint, Christian Hansen, Christian dos Santos Ferreira, Maren Walter, Gustavo Macedo de Paula Santos, Wolfgang Bach

**Affiliations:** 1 MARUM - Center for Marine Environmental Sciences, University of Bremen, Leobener Straße 8, 28359 Bremen, Germany; 2 Faculty of Geosciences, University of Bremen, Klagenfurter Straße 2-4, 28359 Bremen, Germany; 3 Norwegian Offshore Directorate (NOD), Professor Olav Hanssens vei 10, 4021 Stavanger, Norway; 4 Institute of Environmental Physics, University of Bremen, Otto-Hahn-Allee 1, 28359 Bremen, Germany

**Keywords:** Ocean sciences, Solid Earth sciences

## Abstract

Oceanic spreading centers north of Iceland are characterized by ultraslow spreading rates, and related hydrothermal activity has been detected in the water column and at the seafloor along nearly all ridge segments. An exception is the 500-km-long Knipovich Ridge, from where, until now, no hydrothermal vents were known. Here we report the investigation of the first hydrothermal vent field of the Knipovich Ridge, which was discovered in July 2022 during expedition MSM109. The newly discovered hydrothermal field, named Jøtul hydrothermal field, is associated with the eastern bounding fault of the rift valley rather than with an axial volcanic ridge. Guided by physico-chemical anomalies in the water column, ROV investigations on the seafloor showed a wide variety of fluid escape sites, inactive and active mounds with abundant hydrothermal precipitates, and chemosynthetic organisms. Fluids with temperatures between 8 and 316 °C as well as precipitates were sampled at four vent sites. High methane, carbon dioxide, and ammonium concentrations, as well as high ^87^Sr/^86^Sr isotope ratios of the vent fluids indicate strong interaction between magma and sediments from the Svalbard continental margin. Such interactions are important for carbon mobilization at the seafloor and the carbon cycle in the ocean.

## Introduction

Since the first discovery of hot vents and their manifestations at the East Pacific Rise and the Galapagos spreading center^1,2^, hydrothermal systems have been found on nearly all mid-ocean ridges (MOR) over the past 45 years^3^. In general, the high heat flow of the magmatism at the spreading ridges heats up the circulating seawater, which dissolves numerous components from the rocks and evolves into a fluid highly enriched in elements^4^. As the fluid moves from the seafloor into the water column, various precipitates are formed and highly specialized chemosynthetic faunal assemblages become established. The specific formation of such hot venting systems depends on various local factors, such as spreading rates, rock types, tectonic framework, depth of the magma source, heat flow and water depth. While hydrothermal research initially focused on MORs with fast and superfast spreading rates (> 8 cm/year), investigations of fluid venting have progressed towards MORs of intermediate (5–8 cm/year) and slow-spreading rates (2–5 cm/year), because hot vents there are geochemically more diverse (e.g.^5,6^). An even greater challenge was the investigation of MORs with ultraslowly spreading segments (< 2 cm/year) due to the presence of highly segmented rift valleys with complex and rugged terrain, sediment-filled segments, and only sparse young volcanic features. Examples of ultraslowly spreading MORs are the Southwest Indian Ridge^7^ and ridge systems in the Norwegian-Greenland Sea and the Arctic Ocean^8^.

**Figure 1:**
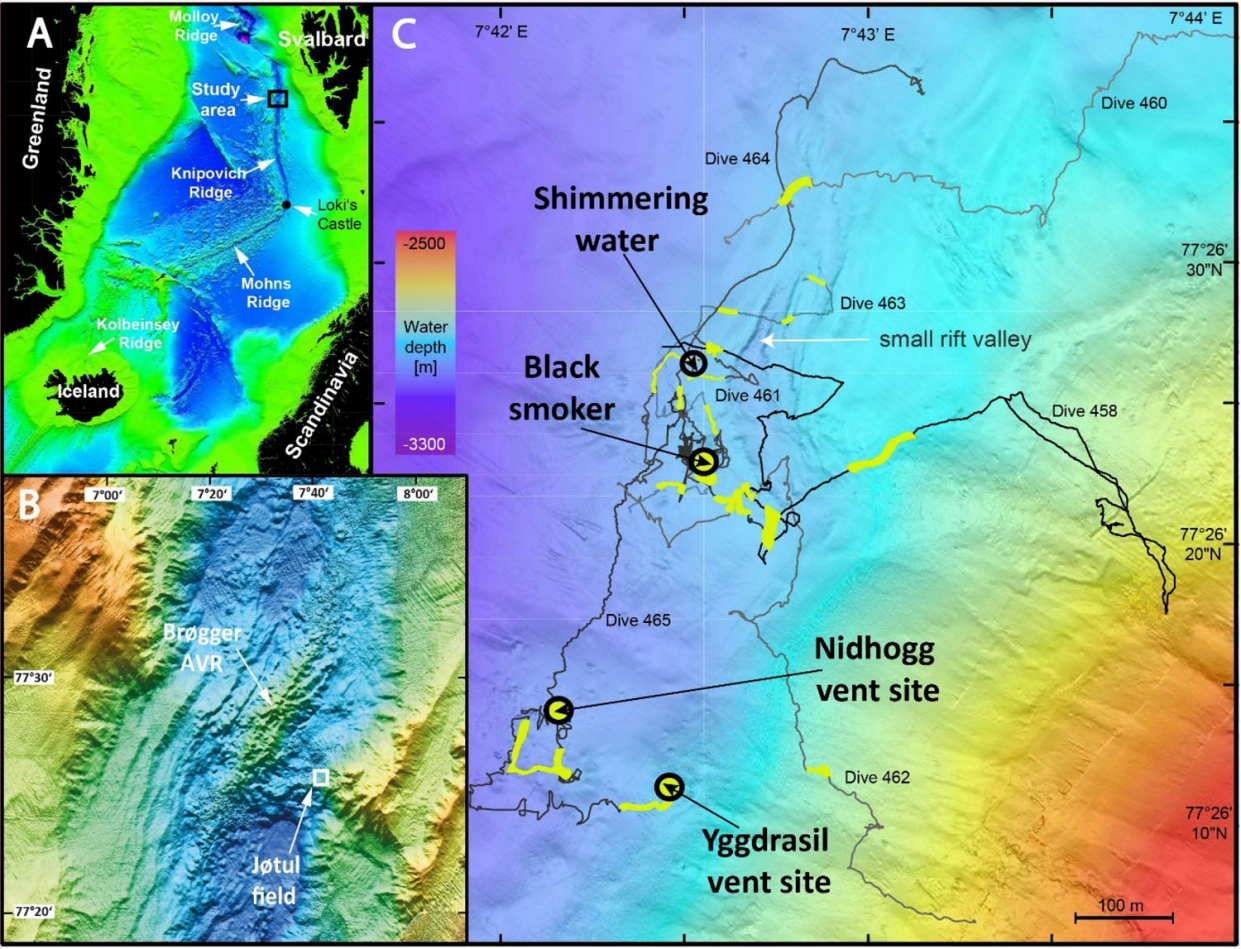
(**A**) Map of the Norwegian-Greenland Sea (GEBCO data) with locations of active seafloor spreading centers and the study area. (**B**) Detailed map of the study area (ship-based multibeam data acquired during cruise MSM109) including the Brøgger Axial Volcanic Ridge (AVR) and the newly discovered hydrothermal active area called Jøtul hydrothermal field. **(C)** AUV-based bathymetry of the Jøtul hydrothermal field (data acquired during cruise MSM109 and provided by the Norwegian Offshore Directorate). Track lines of ROV dives are shown and track portions, where hydrothermal activity was visually observed, are marked in yellow. Four sites were sampled for fluids during MSM109 and are indicated by circles.

The Arctic Mid-Ocean Ridge (AMOR) represents the 4000-km-long ridge system north of the Arctic Circle at 66° N^8^ and comprises several segments of spreading centers separated by distinct fracture zones up the Laptev margin, where spreading terminates at the Lena Delta^9^. Spreading rates appear to decrease from 2 cm/year at the Kolbeinsey Ridge north of Iceland^10^ to less than 1.25 cm/year at the Gakkel Ridge of the Arctic Ocean^11^. The 500-km-long Knipovich Ridge is a prominent rift segment of the AMOR, which approaches the Svalbard continental slope towards the north (Fig. 1A).

### The Jøtul hydrothermal field

Hydrothermal vents associated with the AMOR spreading zone have been studied at Kolbeinsey Ridge^12^, Mohns Ridge^8^, Lena Trough and Molloy Ridge^13^, and Gakkel Ridge^14,15^, while hydrothermal vents at Knipovich Ridge are not yet known. Although signals from hydrothermal plumes have been identified in three different regions, the search for hydrothermal activities at the seafloor during several diving expeditions^16,17^ has remained unsuccessful until now.

During R/V *Maria S. Merian* expedition MSM109, we discovered and sampled a previously unknown vent site at 77° 26′ N on the Knipovich Ridge. Based on information and data collected by the University of Bergen, the Norwegian Offshore Directorate (NOD) conducted an AUV campaign in the Knipovich Ridge area in 2021. The AUV survey data from the Norwegian Offshore Directorate indicated an Eh-anomaly in the area, which guided our planning of the ROV dive locations.

The newly discovered Jøtul hydrothermal field includes black smoker-type venting at T > 316 °C, and venting of clear waters at temperatures between 8 and 272 °C in water depths around 3020 m^18^. Unlike Loki’s Castle at the bend of Mohns Ridge to Knipovich Ridge^19^ (Fig. 1A), the Jøtul hydrothermal field is not related to the Axial Volcanic Ridge (AVR), but is located 5 km east of the crest of the nearby Brøgger AVR (Fig. 1B) at the eastern end of the central rift graben and near the valley-bounding fault. One reason for this peripheral location might be the special tectonic structure of the ridge with its oblique spreading character and the non-transform discontinuity, which are indicated in the bathymetry of the seafloor south of the hydrothermal field (Fig. 1B). Here we report first results from ROV dive observations, vent fluid and precipitate analysis of the Jøtul field, which provide new insights into what governs hydrothermal processes at the ultraslow-spreading Knipovich Ridge.

## Results and discussion

### Hydrothermal plume detection

**Figure 2:**
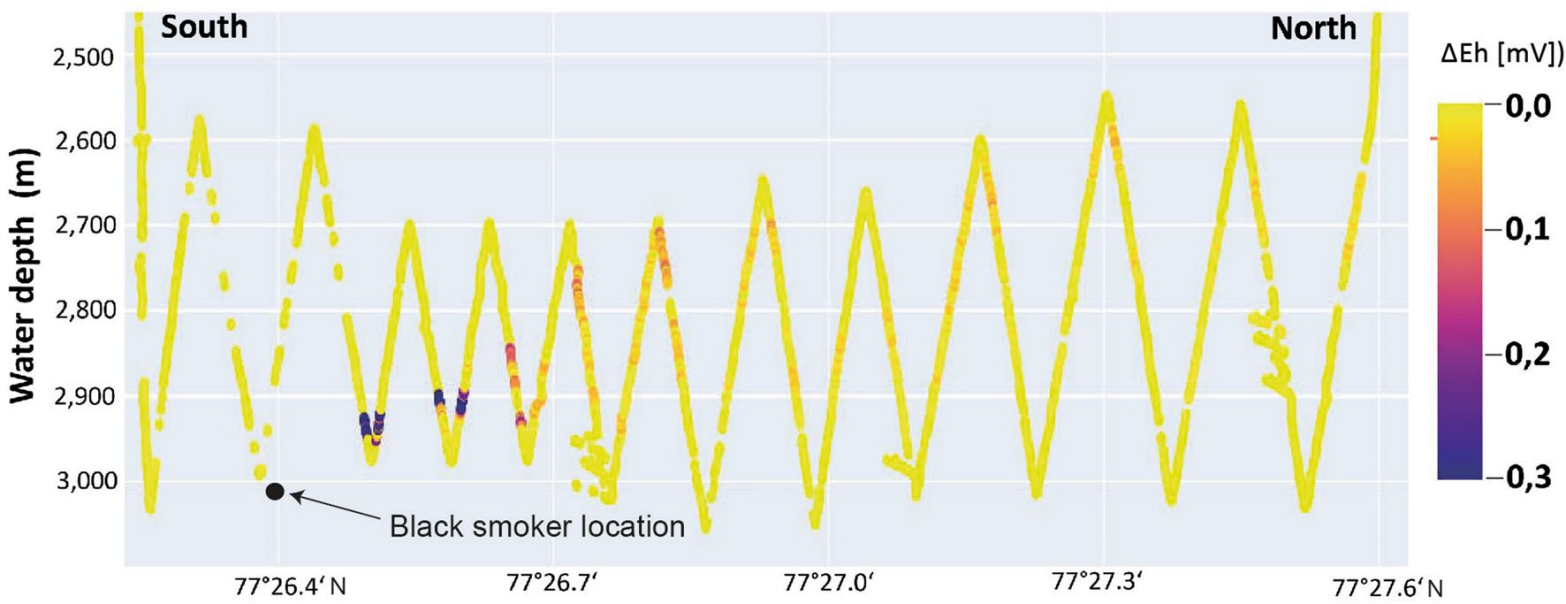
South-North plot of oxidation–reduction potential against water depth over Jøtul hydrothermal field. Data were recorded in mV (ORP sensor) along tow-yo CTD-15, which shows an example of the water column survey. The distribution of higher Eh values show how the hydrothermal plume water is rising upwards and drifting northwards from a black smoker at the seafloor. The drifting hydrothermal signal, documented by a clear weakening of ΔEh values in a northward direction, is consistent with ROV observations of a strong northward flow of bottom water^17^.

**Figure 3:**
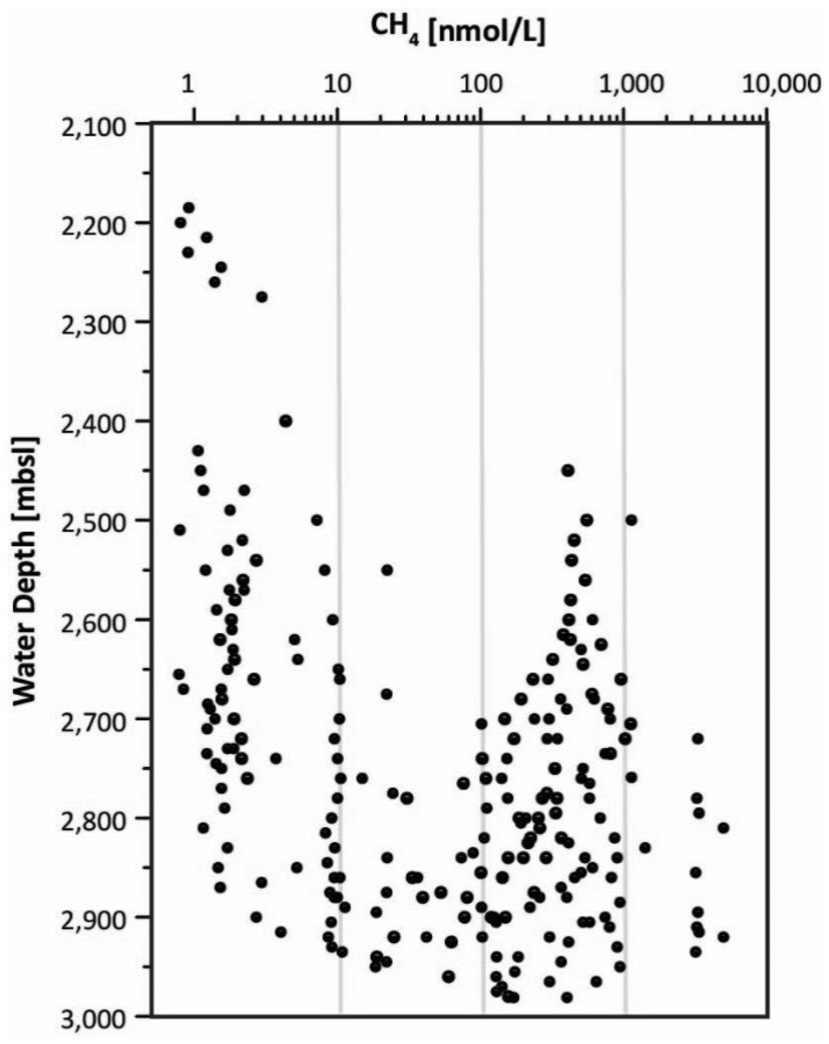
Concentrations of dissolved methane (onboard measurements) in water samples from the Jøtul hydrothermal field and its surroundings plotted against water depth. Samples were acquired during seven stationary CTD casts and four tow-yo CTD profiles during MSM109.

During research expedition MSM109 seafloor exploration using the Remotely Operated Vehicle ROV QUEST discovered hydrothermal activity after hydrothermal anomalies had been detected in the water column. Using a CTD with Niskin water samplers, the near-bottom sea water parameters were investigated along four 2.7–4 km long, N–S and E–W trending towed, yo-yoing (tow-yo) profiles in a see-saw pattern between 2500 and 3200 m water depth^17^. In addition to the usual sensors (conductivity, temperature, pressure and turbidity), an ORP (oxidation–reduction potential) sensor detected changes in the Eh value (ΔEh) indicative of hydrothermal input (Fig. 2). The live ORP data therefore allowed identification of suitable depths for water column sampling with the 23 Niskin bottles. Combined with samples from stationary CTD casts, the tow-yo samples were used to determine the concentrations of dissolved methane close to the Jøtul field area in water depths between 2100 and 3000 m (Fig. 3). Dissolved methane concentrations around 10 nmol/L and less likely represent background values, whereas values around 100 or 1000 nmol/L are related to emissions from strong seafloor sources as reported from hot vents and cold seeps (e.g.^8,20^). Some measurements showed values exceeding 3000 nmol/L, with a maximum of 4991 nmol/L. They represent concentrations that exist very close to the seabed source in seawater that is otherwise highly undersaturated in methane. High methane values occur up to a depth of 500 m above the seabed around the Jøtul Field, documenting the drift dynamic, which is primarily thermally driven.

Hydrothermal input from the seabed is also evidenced by the δ^3^He values because Helium-3, due to its mantle source, serves as a tracer for hydrothermal activity^21^. While, δ^3^He values fall within the range of 7–7.5% in water depths below 2500 m around the Knipovich Ridge^22^, our values aside from similar background values, varied between 20 and 55% in deep water, and were thus much higher in our measurements than in other studies around ultraslow-spreading MORs. Accordingly, the δ^3^He proves not only intense hydrothermal activity, but also the discharge of primordial ^3^He, derived from mantle degassing and mixing with the hydrothermal fluids^23^ at the hydrothermal vents of the Jøtul field.

### Seafloor characteristics and Jøtul vent sites

Guided by unmistakable plume signatures of the hydrothermal vents in the water column, we conducted a total of seven dives with ROV QUEST to record the fluid emission sites, their manifestations at the seafloor, and their distribution within the area (Fig. 1C).

**Figure 4:**
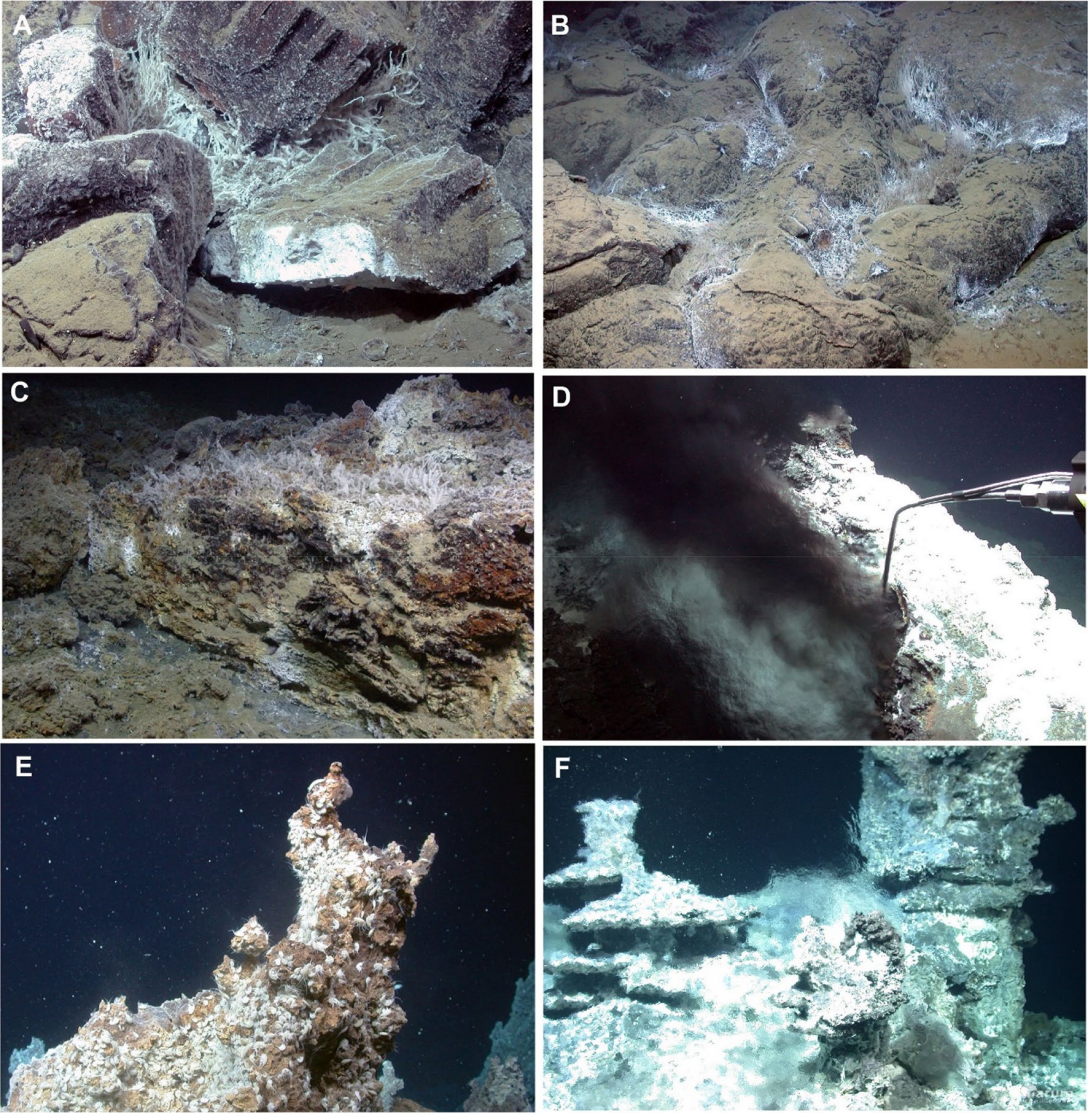
(**A**) Low-temperature vent sites associated with magmatic host rocks. Hydrothermal activity between volcanic blocks with chilled columnar joints is documented by white precipitates of mostly barite, white bacterial filaments and siboglinid tube worms. Small limpets are attached to the white surface in the front and below the rock. (**B**) Sediment-dusted pillow lava flow with indications of hydrothermal venting from in between the lava tubes. (**C**) Seafloor outcrop of mineral precipitates dominated by dolomite, partly associated with siboglinid tube worms covered with white microbial mats. (**D**) Sampling of hot (316 °C) fluids at the black smoker through the intake nozzle of the KIPS sampling device. The sulfide chimney, composed of chalcopyrite, sphalerite, pyrrhotite and anhydrite did not grow vertically, but leans over to the north. This can be explained by bottom-water currents deflecting the outflowing fluid from a vertical to a northerly direction. (**E**) Barite and amorphous silica-rich chimney at Nidhogg venting edifice, densely populated by amphipods. (**F**) Top-most region of Yggdrasil vent site showing multi-flanged precipitation structures and shimmering water.

During the dives mainly seabed structures between 3050 and 2980 m water depth were examined along the foot of the eastern rift graben wall, which, in this area, has an approximately northeast-southwest trending direction. Three of the dives (Dives 458, 460 and 462) were conducted upslope in an easterly direction to water depths as low as 2700 m, but were discontinued due to a lack of observed hydrothermal activity. The dive videos were used to map out all indications of hydrothermal activity along the dive tracks (Fig. 1C). Most of the dives were concentrated around a black smoker, which was found during the exploration phase of ROV Dive 463 (Fig. 1C). Low temperature venting was indicated by several white patches showing fine pipes of siboglinid tube worms intensely surrounded by microbial filaments explored during dive 461 (Fig. 4A,B). Measurements close to numerous locations on bacterial mats showed ambient water temperatures to be only a few tenths of a degree higher than the bottom water, the temperature of which was around 0.6 °C. We could, however, measure a temperature of 8 °C in shimmering water emitted from a fissure (Table 1). XRD analysis of whitish precipitates from this location revealed amorphous silica and barite as the main mineral components.

**Table 1:** Hydrothermal fluid emission sites sampled during MSM109 with temperature and dominant minerals of precipitates.

GeoB Nr	ROV dive	Location	Water Depth (m)	Latitude Longitude	Fluid temperature (°C)	Minerals of precipitates
25024-2	461	Shimmering water	3016	77° 26.444′ N 07° 42.537’E	8.0	Barite, amorphous silica
25031-2	464	Black smoker outflow	3011	77° 26.383′N 7° 42.586′ E	316.6	Chalcopyrite, ISS sphalerite, cubanite, anhydrite
25034-3	465	Nidhogg vent	3031	77° 26.196′ N 07° 42.182′ E	33.6	Barite, amorphous silica, chalcopyrite
25034-8	465	Yggdrasil vent	2989	77° 26.176′ N 07° 42.405′ E	272.0	Pyrrhotite, sphalerite, cubanite, anhydrite

The hydrothermal precipitates at Jøtul are composed of sulfide, sulfate (anhydrite and barite), amorphous silica and carbonate (calcite and dolomite).

The dives showed a smooth seafloor with predominantly soft sediments. Nevertheless, basalt pillows were also encountered in some areas, in particular around a ~ 200-m long, SW–NE trending rift valley (see Fig. 1C). The valley is only 5 m deep in the north, but deepens southwards to about 15 m, before widening to 40 m and changing to a NW–SE strike direction. During Dives 461 and 463 the ROV moved over this graben several times, revealing that the latter's steep walls consist of stacked pillow basalts. The pillow basalts are clearly mid-ocean ridge type (MORB). Indeed, Ti/Y and Zr/Nb ratios of the basalts suggest they are transitional between N-MORB and E-MORB. Black smoker-type hydrothermal activity with a fluid temperature of 316 °C was found west of the wider section of the graben (Fig. 4D, Table 1), but additional hydrothermal areas of diffuse low-temperature venting could also be located on both valley shoulders.

**Figure 5:**
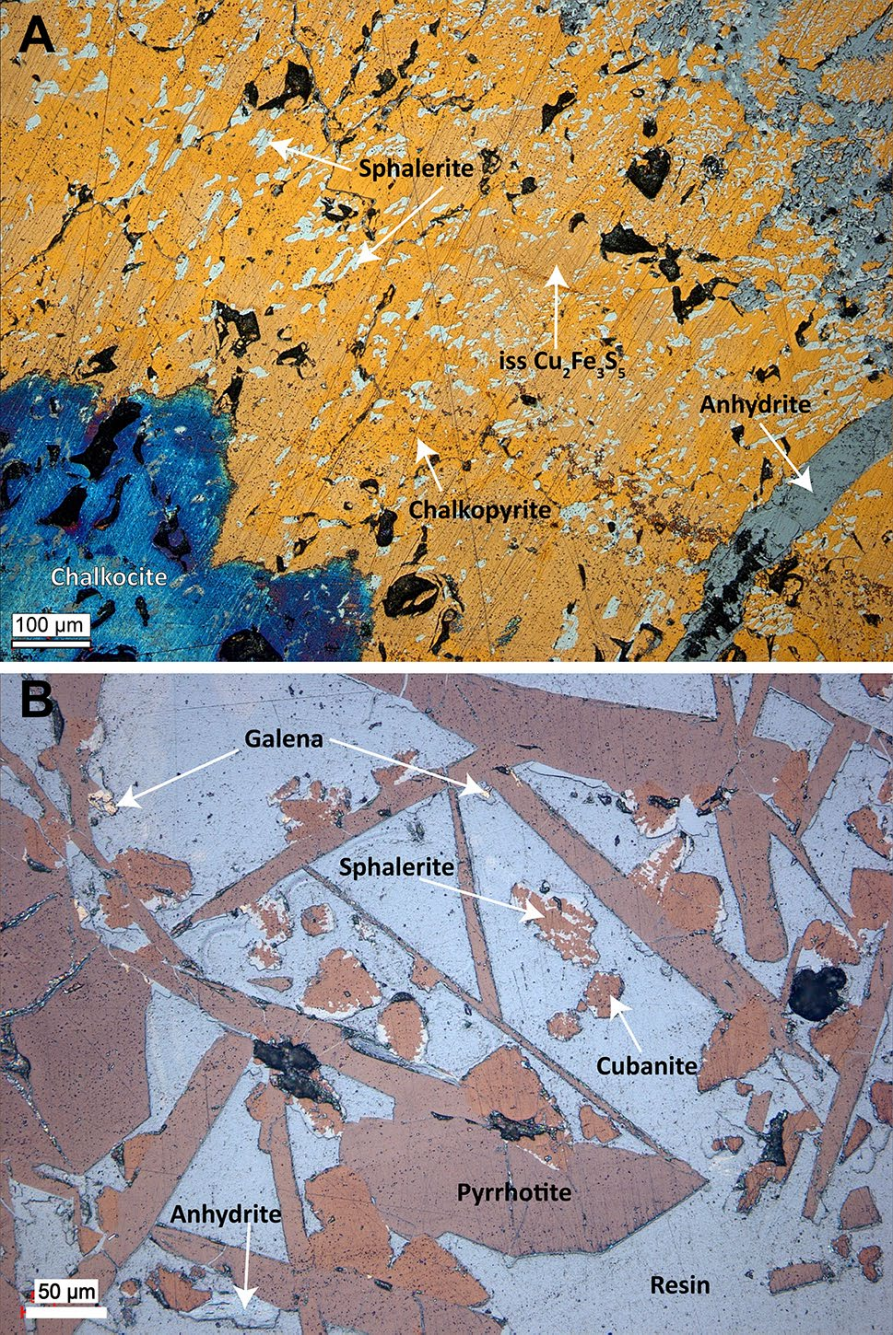
Thin section images of precipitates from Jøtul hydrothermal field taken under reflected light. (**A**) Thin section part of a copper-rich domain typical for the black smoker chimney (GeoB25031-2). Chalcopyrite and ISS are tightly intergrown and co-occur with cubanite and sphalerite. Some of the FeCu-sulfide grains are covered with chalcocite (lower left corner). Anhydrite occurs in veins and as void fill. (**B**) Typical paragenesis of blade-like, pseudohexagonal pyrrhotite crystals with cubanite and Fe-rich sphalerite in a thin section sample of the chimney structure at Yggdrasil vent site (GeoB25034-8), location shown in Fig. 1C. Small grains of galena appear to have formed coevally with sphalerite.

The black smoker was found during Dive 463 and re-visited during Dives 464 and 465 to carry out an extensive sampling program. It is located on the edge of a ~20-m-wide and 6 to 8-m-high mound that is composed primarily of debris from hydrothermal precipitates. The mound shows low-temperature diffuse venting in some places (Fig. 4C) with carbonate precipitates (dolomite and calcite). Thin section analyses of the smoker chimney (Fig. 5A) revealed that it is composed of Fe–Cu sulfide, mainly chalcopyrite (CuFeS_2_), cubanite (CuFe_2_S_3_), and a phase with intermediate composition (Cu_2_Fe_3_S_5_, ISS = intermediate solid solution). Late chalcocite has formed on the surface of the polished slides (Fig. 5A). Also abundant are sphalerite and anhydrite, while pyrrhotite is a minor phase. The sphalerite in all samples has high contents of Fe (around 20 mol%). The mineralization with pyrrhotite, cubanite, and Fe-rich sphalerite is indicative of rather low sulfur fugacities, which can be explained by high ratios of H_2_ to H_2_S in the venting fluids (H_2_(aq) + ½ S_2_(g) = H_2_S(aq)), however, these gases were not measured onboard the vessel. The occurrence of carbonate phases along the commonly observed sulfate minerals is due to the high alkalinity and the high pH value of the vent fluids.

AUV exploration located several additional mounds to the south of the black smoker, two of which also exhibited active hydrothermal venting. The mound at the Nidhogg vent site (Fig. 1C), at a water depth of 3031 m and 300 m southwest of the black smoker, is 10 m high and 30 m across. It appears to be composed of fallen hydrothermal chimneys and infilling hydrothermal precipitate, which XRD measurements showed to consist of predominantly barite and amorphous silica alongside some chalcopyrite traces. Several small chimneys are situated at the peak of the mound, one of which was intensively inhabited by amphipods (Fig. 4E). They serve as outlets of shimmering water, which, at one location, were measured to be at a temperature of 33 °C (Table 1).

The mound at the Yggdrasil vent site, which is situated about 140 m southwest of the Nidhogg vent site (Fig. 1C), is 30 m in diameter and 7 m high. Numerous chimneys and flange-structures on top of the mound issue vigorous flow of clear, shimmering fluids (Fig. 4F), which exhibit temperatures up to 272 °C, i.e. significantly higher than at the Nidhogg vent site. Precipitates at the Yggdrasil vent site consist of pyrrhotite, Fe–Cu sulfides, sphalerite, galena, and anhydrite (Fig. 5B, Table 1).

### Composition of hydrothermal fluids

**Figure 6:**
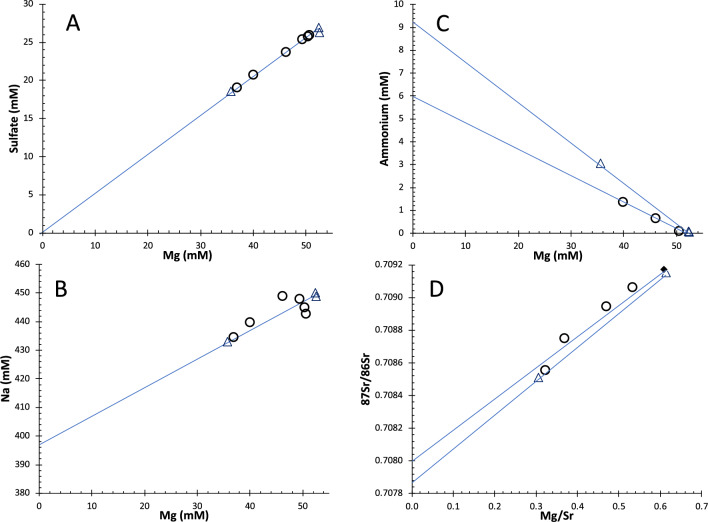
Selected chemical parameters of the Jøtul hydrothermal vent fluids. Circles are for the black smoker fluids and triangles represent fluids from Yggdrasil vent site. Note that the endmember fluid compositions are zero-Mg and zero-sulfate (**A**), and Na is depleted relative to seawater (**B**). Ammonium concentrations are strongly enriched (**C**) and ^87^Sr/^86^Sr ratios (**D**) of the endmember fluids are radiogenic (0.708) relative to most vent fluids (0.704).

The vent fluids are likely zero-Mg, zero-sulfate fluids typical for mid-ocean ridge vent systems^24^. It is hence appropriate to calculate zero-Mg endmember compositions of the vent fluids, which has been carried out for the fluids from the black smoker and Yggdrasil vent site (Fig. 6A; Tables 1 and 2). The fluids from both sites are generally very similar to each other, with the exception of the Fe concentration, which is more than twice as high in the black smoker fluid than in the clear fluid from Yggdrasil vent site. Otherwise, all measured fluids are slightly depleted in chloride compared to seawater, but enriched in Ca, Sr, Si, Fe, etc., which is indicative of intense water–rock reactions. Sodium is more depleted than chloride (Na/Cl = 0.77 vs. 0.84 in seawater) and points to albitization in the reaction zone, where the composition of the fluids is set by water–rock interactions (Fig. 6B). High ammonium concentrations of 9.4 mM in the fluids (Fig. 6C, Table 2) are a clear implication for a very strong presence of sediments in the sub-seafloor reaction zone^25^ and can also explain the relatively high pH of the endmember fluids (Table 2). Likewise, high ^87^Sr/^86^Sr ratios of around 0.708 (Fig. 6D) attest to a radiogenic source of Sr, likely from continental crust material in neritic sediments. A sediment-hosted hydrothermal system for the two sites is further implied by low metal concentrations, similar CO_2_ and CH_4_ concentrations to those in fluid samples from other sediment-hosted hydrothermal vents like Loki’s castle and Guaymas, and overall comparability with vent fluids from Loki’s Castle (Table 2). Conversely, basalt-hosted hydrothermal systems, like Lucky Strike, often have higher carbon dioxide concentrations and significantly lower methane concentrations than those observed in the Jøtul fluids (Table 2)^26^. Headspace gas samples from the latter show molecular hydrocarbon ratios (C_1_/C_2_ + C_3_) between 107 and 117, δ^13^C-CH_4_ values of − 30.5 to − 29.7‰, and δ^2^H-CH_4_ values of − 107‰ (Table 2), all of which are typical indications for thermogenic methane generated under higher temperatures^27^. Similar light hydrocarbons were also produced in sediments of the Guaymas Basin^28^, where magmatic intrusions lead to thermo-catalytic degradation of organic matter in sedimentary deposits.

**Table 2:** Calculated endmember (Mg = 0 mM) fluid compositions from Yggdrasil vent site (Sample GeoB25037-8) and Black Smoker (GeoB25031-2) from our own data, compared to endmember compositions from the literature: Loki’s Castle, Guaymas (sediment-hosted^6^), and Lucky Strike (basalt-hosted;^6^).

		Yggdrasil vent site	Black smoker	Loki’s castle	Guaymas	Lucky strike
T	[°C]	272	316	317	315	305
pH	@25 °C	5.3	5.2	5.7	6	4
NH_4_	[µM]	9359	7320	5040	14,800	9
Mg	[mM]	0	0	0	0	0
Cl	[mM]	509	530	490	598	489
Na	[mM]	394	399	396	483	385
Ca	[mM]	37.4	37.3	27.9	30	35.7
Si	[mM]	13.6	14.8	15.5	12.8	15.4
Fe	[µM]	32.7	75	26	41.5	435
Sr	[µM]	177	175	99	195	93
^87^Sr/^86^Sr		0.7079	0.7080	0.7082	0.7055	0.7043
CO_2_	[mM]	26.4	25.5	24.5	50	64
CH_4_	[mM]	9.5	8.8	13.9	59	0.7
δ^13^C-CH_4_	[‰]	− 30.5	− 29.7	− 27.9	− 47	− 13.5
δ^2^H-CH_4_	[‰]	− 107	− 107	− 111	− 196	

The full data set of all measurements from Jøtul field will be stored in the P ANGAEA data bank.

## Conclusions

Although ultraslow spreading ridges represent a quarter of all spreading ridges globally, very little is known about their hydrothermal systems. The Jøtul hydrothermal field is the first to be discovered along the 500-km-long ultraslow-spreading Knipovich Ridge and is significant, because it represents a new link between the active hydrothermal systems of Loki's Castle at the bend of Mohns and Knipovich Ridges^18^ and the Aurora hydrothermal field of the Gakkel Ridge^14^. Since these systems are separated by a distance of more than 1000 km, the discovery of the Jøtul hydrothermal field is important for the understanding of chemosynthetic faunal community distribution^29^. Furthermore, unlike in other hydrothermal fields at ultraslow spreading ridges, where volcanic activity is particularly linked to AVRs (e.g. Lokis’s Castle^18^), the Jøtul hydrothermal field is actually located ~ 5 km west of the Brøgger AVR. This location at the eastern boundary fault of the Knipovich rift valley coincides with the slope aprons of the Svalbard continental margin. One reason for the peripheral location of the Joetul field in the Knipovich rift graben is possibly the special tectonic structure of the ridge with its oblique spreading character and the non-transform discontinuity, which is indicated in the bathymetry of the seafloor south of the hydrothermal field (Fig. 1B). Based on the observed fluid chemistry and paragenesis of the hydrothermal precipitates at both black smoker and Yggdrasil sites, the circulating fluids likely had an intensive exchange with very thick sediment deposits^30^. This, in turn, suggests an intense interaction of some ridge magmas with the Plio-Pleistocene sediments of the Svalbard continental margin. During this exchange, a large amount of thermogenic methane is formed, which could be detected in high concentrations in the plume of the Jøtul field. Our study therefore shows that high hydrocarbon seafloor emissions generated by hydrothermalism are not only related to sediment-covered basins such as the Guaymas Basin^31^, the Okinawa Trough^32^, or the Escanaba Trough^33^, but can also to be expected along narrow ultraslow-spreading ridge systems such as the Knipovich Ridge.

## Methods and samples

Scientific field work was carried out on board the research vessel *MARIA S. MERIAN* during cruise MSM109 in July 2022^17^ and was supplemented by subsequent laboratory work. Data presented here include acoustic measurements, water column data and sampling surveys with the MARUM ROV QUEST.

### Multibeam AUV and ship-based data

The bathymetric overview mapping of the Knipovich central graben and its flank areas was carried out with the hull-mounted Kongsberg EM122 multibeam. The EM122 MBES is a well‐known deep‐sea system operating with 11.25–12.5 kHz. Onboard R/V *MARIA S. MERIAN*, a beam width configuration of 2° (TX) by 2° (RX) is installed and a swath angle of up to 150 degrees can be reached with a maximum coverage of 5.5 times the water depth. During cruise MSM109, the maximum swath width was set to 130° to improve data quality, reduce the amount of noisy data at the outer beams, and increase the ping rate. Sound velocity profiles were calculated from CTD data and applied to the raw data during acquisition via the SIS software from Konsberg. The EM122 bathymetry data were processed with the open-source software package MB‐System^34^.

For high-resolution seafloor mapping, the autonomous underwater vehicle MARUM SEAL5000 was used. The vehicle is 5.75 m long, 0.73 m in diameter and has a weight of 1.35 tons (https://www.marum.de/en/Infrastructure/MARUM-SEAL.html). The AUV is equipped with the Kongsberg EM2040 multibeam and can be used with three different frequencies: 200, 300, and 400 kHz. 158 km track lines were surveyed during five dives within the Jøtul field area. Three surveys were conducted with a frequency of 200 kHz and with a line spacing of 300 m at an altitude of 130 m above seafloor. Two additional dives were conducted for surveys in 60 m altitude using 400 kHz frequency, which results in an even higher resolution of the target area. The data were also processed with the software package MB-System and merged with an AUV-derived data set from Notwegian Offshore Directorate (2021-NPD-01). The latter data were acquired with an AUV carrier using the Konsberg EM2040. (https://npd.maps.arcgis.com/apps/webappviewer/index.html?id=cfc3c31304fe4eb8974b7d3a4bbf8c4d) during a cruise with the vessel Olympic Delta in 2021.

### Seabed survey and sampling

For vent search and near-seafloor investigation, the deep water ROV (remotely operated vehicle) MARUM-QUEST 4000 m was used. Several cameras mounted at different positions of the ROV allow for a very good overview of the illuminated areas on the seabed. Operation of specific payload devices and the sampling of rocks, precipitates, fluids, and sediments was done via two hydraulic manipulators. Fluid samples were sucked in via the pump of the KIPS sampler from a mobile titanium nozzle held by the manipulator arm. Within the KIPS system the fluids were filled into special sample containers. A temperature sensor attached in parallel to the opening of the nozzle guided the pilots to the hottest place of the fluid emission sites. For GPS-based positioning, the shipboard Sonardyne Ranger USBL system was used. Seven dives were performed within the Jøtul hydrothermal vent field and its surroundings.

### Water column work

To detect hydrothermal plumes in the water column an ORP (oxidation–reduction potential) sensor^35,36^ provided by the Pacific Marine Environmental Laboratory (PMEL, Seattle, WA, USA) was used. Values of the ORP (in MV) immediately decrease, when nanomolar concentrations of reduced components are detected in the water column. These may include hydrothermal fluid ingredients (e.g., Fe^2+^, HS^−^, H_2_) that are out of equilibrium with oxidizing seawater. Signal recovery of the ORP sensor is slower than the initial response, depends on the compound detected, and may take several tens of minutes, so we followed the criteria proposed by Baker et al.^36^ for identification of ORP anomalies: a sustained negative gradient (d*E*/dt < − 0.02 mV s^−1^) for consecutive measurements and an overall decrease of ΔE ≥ 1 mV. The online ORP sensor showed live changes in the redox potential of the water column during the deployment of the CTD/rosette. Each time a signal of a plume was detected with the ORP sensor, a bottle of the CTD/rosette water sampling systems was closed to sample the water from the exact position.

The CTD/rosette including 23 10-L Niskin water samplers. During the MSM109 cruise, ten CTD/rosette stations were conducted within the Jøtul hydrothermal field, four of which were so-called tow-yo stations^17^. During a single cast, the CTD/rosette-system was lowered with a speed of 0.5 m/s. After the CTD/rosette-system reached a near-seafloor depth, the samplers were successively closed during up-cast every 15–20 m for the deepest few hundred meters. During tow-yo casts, the vessel was moved continuously at a speed of 0.5 knots along a designated track, while the CTD/rosette was repeatedly lowered and heaved between the seafloor and 300–500 m above it at a speed of 0.5 m/s. The four tow-yo CTD/rosette casts were carried out along 2.7–4-km-long profile sections in water depths between 2500 and 3000 m to sample and examine the near-bottom water column.

### Dissolved methane and helium isotopes in the water

All water samples were analyzed for dissolved methane concentrations. Preparation of water samples followed the procedure described in detail in Mau et al.^37^. 100 mL of water each were transferred from Niskin bottles into two 140-mL plastic syringes immediately after recovery of the CTD/rosette system on deck, and great care was taken to avoid the entry of gas bubbles into the syringes. A 40-mL headspace gas volume was generated in each syringe with methane-free air and the headspace volume from both syringes were combined. Methane concentrations in the headspace gas were measured on board using a Greenhouse Gas Analyzer (GGA-30r-EP; Los Gatos Research, California, USA).

17 selected water samples were taken from hydro-casts of CTD/rosette stations for the determination of noble gas isotopes (^3^He, ^4^He, ^20^Ne, ^22^Ne). The sampling was performed directly from the CTD/water bottle rosettes without contact to atmospheric air into 40 mL gas tight copper tubes, which are clamped off at both sides. To avoid contamination, they were collected from the rosette bottle before any other water sample. For each sampling, great care was taken to debubble the plastic sampling tube by allowing the water to flow for as long as needed and periodically tapping the copper tube with a wrench. The analysis of samples was done in the lab of the Institute of Environmental Physics, University of Bremen (IUP). The samples were connected to a fully automated UHV mass spectrometric system equipped with a two-stage cryogenic system and a quadrupole and a sector-field mass spectrometer. The system is calibrated regularly with atmospheric air standards (reproducibility < 0.2%). Measurement of line blanks and linearity are done as well. The performance of the Bremen facility and the procedure is described in Sültenfuß et al.^38^. The ^3^He and ^4^He isotopic ratios are reported as δ^3^He, the excess of ^3^He compared to the atmospheric equilibrium in %. The precision of He is 0.4%.

### Hydrothermal fluids

Hot, focused and diffuse fluids were sampled with the fully remotely controlled flow-through fluid sampling system KIPS (Kiel Pumping System, KIPS-4^39^) made entirely of inert materials, such as perfluoralkoxy (PFA) and high-purity titanium (four bottles, 750 mL each). During ROV-based sampling, fluids enter the KIPS via a titanium tube, that is guided by the ROV’s manipulator to the point of sampling. Sampled fluids are pumped through coiled PFA tubing to the remotely controlled valve pack with four sample lines and one purge line. The gear pump (0–1 L/min) is mounted downstream and an in-line flow meter delivers real-time data for flow rate and total fluid volume. Sample flasks are pre-filled with ambient bottom water and sampled fluids are slowly pumped over five minutes, giving a > fivefold exchange of the flask’s volume. In total, twelve KIPS samples were retrieved during MSM109. Directly upon recovery of the ROV, all KIPS’ samples were divided into different aliquots for the analyses of trace metals, dissolved gases, isotopes and nutrients.

For major, minor, and trace element analysis, the samples were acidified with suprapure HCl to pH ~ 1.7 (final concentration 0.024 M HCl) and stored in acid-cleaned polyethylene bottles at 4 °C until further analysis. All samples were filtered through pre-cleaned 0.45 µm syringe filters and subsequently measured with ICP-MS and ICP-OES at the University of Bremen. Major elements have been measured in the MARUM sediment geochemistry lab with a Varian Vista Pro simultaneous radial ICP-OES equipped with an Ar-gas humidifier, a seaspray nebulizer and a cyclonic spray chamber. Trace element analyses were conducted in 100-fold diluted aliquots using a Thermo Scientific Finnigan Element2 HR-ICP-MS in low, medium and high-resolution mode with indium, bismuth and rhodium as internal standards. Accuracy and precision were checked with international seawater reference materials from the National Research Council Canada (CASS-6, NASS-7 (spiked)) and were within ± 5% of the reference values, except for Uranium (+ 10%).

Ammonium (NH_4_^+^) and total alkalinity (TA) were measured on frozen and thawed samples at the Leibniz Centre for tropical Marine Research with a TECAN infinite M2100Pro microplate spectrophotometer (TECAN Trading AG, Switzerland) following the methodological procedures after Ringuet et al.^40^, Benesch and Mangelsdorf^41^ and Sarazin et al.^42^ with slight modifications resulting in a better resolution for lower concentration (0–55 µM NH_4_^+^, 1500–4000 µM TA).

The preparation and isotope ratio measurements of Sr were carried out in the Isotope Geochemistry Laboratory at MARUM—Centre for Marine Environmental Sciences, University of Bremen. Vent fluids were evaporated to dryness, re-dissolved in HNO_3_ and chemically separated followed the method presented in Höppner et al. (2018)^43^. Strontium isotope ratios were analysed on a TRITON Plus thermal ionization mass spectrometer (ThermoScientific) in dynamic mode. Instrumental mass fractionation during isotope analysis was corrected using the stable ^86^Sr/^88^Sr ratio of 0.1194. The ^87^Sr/^86^Sr ratio of reference material NIST SRM 987 was 0.71024 ± 0.00002 (2sd_mean_, n = 2), which is consistent with the concurrent intermediate reproducibility of 0.710250 ± 0.000015 (2sd, n = 38), and match the range of previously published TIMS-derived values of 0.710250 ± 0.000034 (2sd_mean_, n = 1245, calculated from GeoRem database June 2022; http://georem.mpch-mainz.gwdg.de).

Dissolved gas characteristics for CH_4_ and ΣCO_2_ were quantified from dedicated 250 mL sample aliquots that were filled into septum vials while carefully avoiding trapping any atmospheric bubbles. For determining concentrations, subsamples of approx. 10 mL were drawn from the 250 mL septum vial (while balancing pressure with Helium), transferred into a pre-weighed, He-rinsed and vacuumized 25 mL septum vial and complemented with 0.5 mL 85% H_3_PO_4_ to convert all dissolved inorganic carbon to CO_2_. A syringe was pre-filled with 20 mL He at 1 atm to enable volumetric quantification of over- or under-pressure within the septum vials’ head space. Subsequently, the gas mixture was homogenized and then volatiles were quantified in triplicates using an Agilent 7820 A Gas Chromatograph equipped with a Thermal Conductivity Detector (TCD; ΣCO_2_) and a Flame Ionization Detector (FID; CH_4_) following separation through a packed HaySep 80/100 column (Sigma-Aldrich) at 40 °C with He as carrier gas. Measurements were calibrated with a certified gas mixture (CRYSTAL, AirLiquide) and triplicate measurements were well reproducible (< 1 RSD%). Actual concentrations within the original fluid samples were derived after considering volume proportions (V_headspace_/V_sample_), dilution (20 mL He), and equilibrium between gas and fluid phase (Henry constant).

For analysis of C_1_–C_6_ hydrocarbon compositions and stable carbon and hydrogen isotopic signatures and CO_2_, sampling aliquots were filled into 250 mL-septum and sealed with caps and rubber stoppers. 10 mL of the samples were withdrawn using a syringe and cannula while simultaneously injecting 10 mL of helium to create a headspace. The molecular composition of hydrocarbons was measured by gas chromatography according to Pape et al.^44^. Stable carbon and hydrogen isotopic compositions (^13^C/^12^C; ^2^H/^1^H) of CH_4_ and CO_2_ were determined by GC-isotope ratio mass spectrometry at MARUM.

Since seawater or pore water is always entrained when sampling hydrothermal vent fluid, or is already mixed into the fluid below the seabed the sample is usually a mixture of several sources. In the unaffected hydrothermal fluid, the quantitative removal of Mg is given by the seawater-rock interaction under high temperatures^45^. Therefore, a zero Mg fluid is assumed to represent the unaffected hydrothermal fluid and the endmember concentrations of the measured hydrothermal vent fluids have to be determined. Endmember values of the concentrations were linearly extrapolated by regressing the measured fluid composition and seawater values to zero Mg. Where several samples of a venting fluid were available, all measured values were regressed together to obtain a single endmember.

### Thin sections and mineral analysis

Thin sections of chimney material were inspected by reflected light microscopy. The thin sections were then carbon-coated and mineral compositions were determined using a CAMECA SX-100 microprobe at the Faculty of Geosciences, University of Bremen. Quantitative analyses used a point-focused beam with an acceleration voltage of 20 kV and a beam current of 20 nA. Pyrite, chalcopyrite and sphalerite^46^ were used as reference standards to control analytical precision and quality of the data.

## Data Availability

All fluid chemistry data are stored in PANGAEA (Data Publisher for Earth & Environmental Science, https://doi.pangaea.de/10.1594/PANGAEA.966848 ). Further datasets used during the current study are available from the corresponding author on reasonable request.
